# Japanese encephalitis in Bali, Indonesia: ecological and socio-cultural perspectives

**DOI:** 10.1080/23144599.2021.1975879

**Published:** 2021-09-16

**Authors:** I Made Kardena, Anak Agung Ayu Mirah Adi, Nyoman Mantik Astawa, Mark O’Dea, Michael Laurence, Shafi Sahibzada, Mieghan Bruce

**Affiliations:** a Department of Biopathology, Faculty of Veterinary Medicine, Udayana University, Denpasar, Indonesia; b School of Veterinary Medicine, College of Science, Health, Engineering and Education, Murdoch University, Perth, Western Australia; c Department of Primary Industries and Regional Development, Dpird Diagnostics and Laboratory Services, Sustainability and Biosecurity, South Perth, Western Australia

**Keywords:** Bali, Japanese encephalitis, potential ecological determinants, socio-cultural practices

## Abstract

The increasing number of cases of acute encephalitis syndrome, a key presenting clinical sign of Japanese encephalitis infection in humans, along with increasing laboratory confirmed cases in Bali over recent years have led to the Indonesian government developing a national program of vaccination against Japanese encephalitis virus. In order to inform multidisciplinary management, a review was conducted to assess Japanese encephalitis virus-related cases in humans and animals including their determinants and detection in vectors. Along with published literature, key data from local authorized officers in Bali have been used to convey the recent situation of the disease. Related surveys detected up to 92% of the local children had antibodies against the virus with the annual incidence estimated to be 7.1 per 100,000 children. Additionally, reports on young and adult cases of infection within international travellers infected in Bali were documented with both non-fatal and fatal outcomes. Further seroprevalence surveys detected up to 90% with antibodies to the virus in animal reservoirs. The detection of the virus in certain *Culex* mosquito species and high levels of seropositivity may be associated with greater risk of the virus transmission to the human population. It was also highlighted that local sociocultural practices for agriculture and livestock were potentially associated with the high density of the vector and the reservoirs, which then may lead to the risk of the disease transmission in the ecology of Bali.

## 1. Introduction

Japanese encephalitis (JE) is a potentially fatal, zoonotic viral disease caused by JE virus (JEV) [1]. The virus is grouped within the family *Flaviviridae*, along with other arthropod-borne flaviviruses, including Dengue virus, West Nile virus (WNV), Tick-borne encephalitis virus (TBEV), St. Louis encephalitis virus and yellow fever virus (YFV) [2]. JEV is an enveloped, icosahedral virus, with a diameter of approximately 50 nm, and consists of a single-stranded positive-sense ribonucleic acid genome [3], of approximately 11 kb in length [4]. The open reading frame in the genome encodes a single polyprotein, which is intracellularly cleaved into ten viral proteins, including three structural (envelope/E, pre-membrane/PrM, and core/C) and seven non-structural (NS) proteins (NS1, NS2A, NS2B, NS3, NS4A, NS4B and NS5) [5,6].

JEV exists as a single serotype within which are five genotypes, GI–GV [7–9]. The genotypes of JEV have been characterized based on the nucleotide sequence of the viral envelope (E) protein [10]. The E protein is a major structural protein that contains the receptor-binding domain and the neutralization epitopes. Additionally, Wei et al. [11] also argued that the amino acid variation in the E protein of the JEV may affect virulence and antigenicity.

The transmission of JEV into animals and humans is through mosquito bites. Several genera of mosquitoes can transmit the virus, however, *Culex* spp. are dominant [12,13], primarily *Cx. tritaeniorhynchus* [14,15]. This mosquito vector tends to breed and lay their eggs in irrigated rice paddy fields, associating this farming practice with the increased mosquito populations and subsequent increased risk of JE infection in humans [16].

JEV infection in humans is also associated with the infection in animals, especially pigs and wading birds. Both of the animals have an important role in the ecology of JEV [17]. *Ardeidae* birds, including egrets and herons, are the reservoir hosts of JEV [18,19]. Meanwhile, pigs act as amplifying hosts that producing large amounts of infectious virus during the viraemia phase, resulting in uptake of virus by feeding mosquitoes [20,21]. Most infected mammals and birds are asymptomatic or develop mild clinical signs such as resolving fever and inappetence, but in infected pregnant sows, the infection may result in abortion, stillbirth and congenital deformity [21,22].

Aside from *Ardeidae* birds and pigs, evidence of JEV infection has been reported in horses, dogs, cats, cattle, snakes, frogs, sheep, goats, monkeys, racoons, fresh water turtles and other birds including chickens and ducks [21,23,24]. The majority of these are dead end hosts, although ducks and chickens are suspected to have a role in disease transmission as they appear to develop viraemia to a sufficient titre to infect feeding mosquitoes [25–27].

Although the incidence of Japanese encephalitis disease has decreased globally due to implementation of vaccination programs, the disease is still a public health threat partly as a result of vector expansion due to climate change [8]. The virus is estimated to infect almost 68,000 humans each year [28] with approximately 75% of cases occurring in children and resulting in development of acute encephalitis syndrome (AES) [29]. The case fatality rate for the disease may reach 30%, and among those patients who survive 30–50% may develop long-term neurological sequelae [30]. In addition, the distribution of the vector and virus indicates around three billion people in the world are at risk of the infection [31], spanning countries in Oceania and Asia, including Indonesia [32].

Indonesia is recognized as a part of Indo-Malayan region where JEV is considered to have originated. The first JE infection was reported in the 1970s and the virus was successfully isolated. JE-related cases have been detected in 29 out of 34 provinces, including the province of Bali [13].

In Bali, clinical and confirmed cases of JEV infection in humans were reported in 2014 increasing until early 2018 when the national vaccination program against JEV in humans was firstly implemented in Bali in March 2018 [33]. However, vaccination in humans cannot eliminate the virus in the environment, as JE is a zoonotic disease with multifactorial elements involved in transmission, such as human agricultural activities, animal reservoirs and the mosquito vector interact in a socio-cultural-environmental ecology. This review was conducted to assess JE-related cases in humans, animals and their determinants, and detection in vectors, including socio-cultural practices of the Balinese, which may associate with the potential risk of Japanese encephalitis infection in the area.

## 2. Methods

**Figure1.**
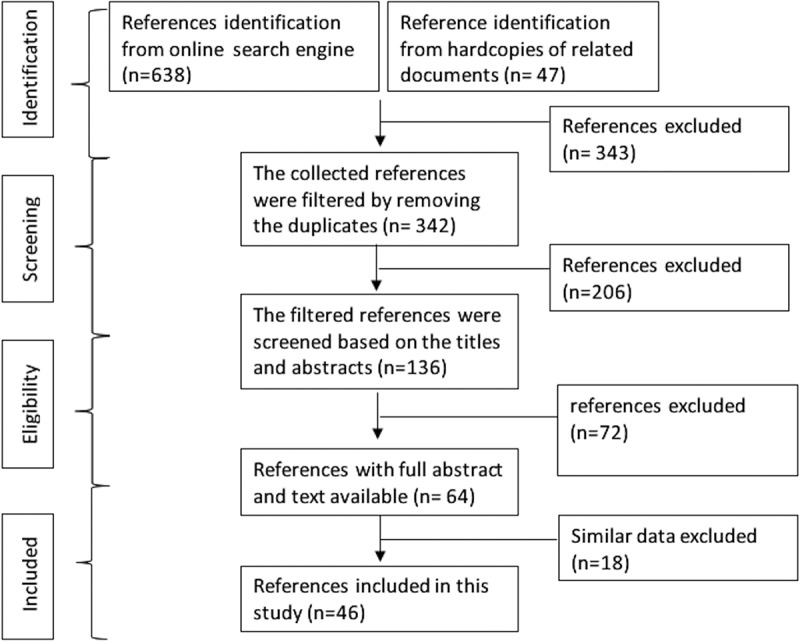
A flowchart of the selection process of related study references used in this review by using preferred reporting items for systematic reviews and meta-analysis (PRISMA)

Published articles on JEV that reported predominantly in Indonesia and more specifically in Bali were reviewed. Online search engines, such as PubMed, Google Scholar, Portal Garuda and Indonesian Publication Index, were the databases that used to find the related articles. Keywords used were “Japanese encephalitis”, ”Flavivirus”, “Indonesia”, “Bali”, “epidemiology”, “distribution”, “risk factors”, “humans”, “vector”, “animals”, “ecology” and “social-culture”. Hardcopies of related documents, including seminar presentations and unpublished online data documents from the Health department of Bali Province, the Agriculture Department of Bali Province, and Disease Investigation Centre (*Balai Besar Veteriner*) Denpasar, were also collected, evaluated and summarized to be used in this review based on preferred reporting items for the systematic reviews and meta-analyses (PRISMA) guidelines conducted in October 2020 to February 2021. The shortlisted articles or documents were filtered and compiled based on the inclusion criteria focusing on the detection cases of Japanese encephalitis in humans, mosquito vector and animals in Bali including the articles on Balinese socio-cultural practices. When the articles did not meet the criteria, they were excluded (Figure 1).

## 3. Results and discussion

Bali is an island province which is located between Java and Lombok islands in the Indonesian archipelago. The island is approximately 5,632 km^2^, inhabited mostly by Balinese who are mainly Hindu. Bali is also recognized as an ecotourism destination due to its agricultural landscape and socio-cultural environment. The local traditions and geographical environment have attracted not only national domestic tourists, but also international travellers to visit the island.

### 3.1. JEV genotypes detected in Indonesia and Bali

Studies on detecting JEV genotypes in Indonesia have been conducted and found four of the five genotypes in the country. A total of 37 isolates gathered between 1974 and 1987 in mosquito and pig samples collected from Indonesian archipelago, the GII, GIII, and GIV were identified [34]. A more recent study found that GI was isolated from *Cx. gelidus* in the province of Jambi, Indonesia [9].

Genotyping studies on JEV in Bali are very limited. To date, GIV is the only genotype found in Bali. In 1980, GIV was isolated from *Cx. tritaeniorhynchus* [34]. Similarly, in 2017, GIV virus was isolated from pig serum samples [35].

### 3.2. Cases and determinants related to Japanese encephalitis in Bali

A number of the JE-related studies in Bali have been established. A cross-sectional study conducted by Kari et al. [36] described that 34% of 158 patients with AES from July 2001 to January 2003 confirmed the evidence of infection by detection of anti-JEV IgM in the patients’ cerebrospinal fluid (CSF). The study also assessed risk factors of the infection in which owning pigs and rice farming were significantly associated with the presence of JE [36]. Similarly, a case-control study demonstrated that people with houses located next to rice paddy fields and living in close proximity to pigs were more than four times more likely to be JEV infected [37]. Another study also identified the same associated factors, although some protective factors included window screens, mosquito bed nets and mosquito repellent were also assessed [38].

#### 3.2.1. Japanese encephalitis-related studies in humans in Bali

In 1970s, a JEV serological survey was conducted to detect the antibodies in some areas of Indonesia, such as Pontianak in which antibody prevalence was 26% (25/94), Samarinda 27% (31/121), Balikpapan 22% (37/172), Surabaya (Java island) 2% (1/50), Lombok 16% (18/115), Kupang 2% (2/98), Ujung Pandang 2% (3/174), Jayapura (West Papua) 3% (4/170), Ambon 5% (6/125) and Bali at 52% (48/94) [39,40]. The same antibody proportion was also demonstrated in a survey performed from October 1990 to July 1995 [41].

A two-series survey in humans in Bali was conducted in 1996–1997 and the antibodies were detected [42]. Next survey conducted in 2001 to 2003 also detected the antibodies with annual incidence rate was higher compared to the global incidence rate [43] with 7.1 per 100,000 children, making the virus in Bali hyperendemic [44]. Ninety-two percent of the antibodies was found in children under 15 years old [45], which were distributed in all of the districts and a city areas based on the data gathered from all of the district level and central hospitals in Bali [36].

#### 3.2.2. Japanese encephalitis infected international travellers in Bali

JEV infection not only affects locals but impacts on Bali’s key tourism market through the effects on international travellers. Cases that have been acquired by travellers to Bali include fatal and non-fatal encephalitis [13,46]. A ten-year-old girl from Australia had been diagnostically confirmed infected by JEV after returning from a two-week holiday in Bali [47]. Similarly, a 60-year-old Swedish woman [48] and an 80-year-old man were suspected to be infected with the JEV after they had a 2-week holiday on the island [49]. A similar case occurred in a 54-year-old Germany woman [50] and a 45-year-old Australian man after having a holiday in Bali [51] and their serological diagnostics detected the antibodies against JEV. However, fatal cases of JE infection were reported occurred in a Danish man who had a 12-day holiday [52] and an 59-year-old Australian male who stayed for three months in Bali [53] after their diagnostic tests confirmed that they were infected with the JEV (Table 1).

**Table 1.** JE-related cases in humans in Bali

Year study	Findings	Test used	References
Cases in locals			
1972	52% of human samples in Bali detected to have the antibodies against JEV.	HI	[40]
1990–1995	Antibodies against JEV detected in 40 out of 77 (51.9%) human samples with acute viral encephalitis collected from Sanglah central hospital, Bali.	ELISA	[41]
1993–1997	Most of human samples collected in Bali (up to 90%) had the antibodies against JEV.	HI	[13,39]
1996–1997	Antibodies against JEV were detected in two series surveys of patients with viral encephalitis.	ELISA	[42]
2001–2003	As many as 86 patients confirmed and 4 probable cases of JE identified with incidence rate at 7.1 per 100,000 children under 12 years old.	ELISA	[44]
2001–2003	A cross sectional study found 34% (55/158) of viral encephalitis patients were confirmed with JE infection. The patient samples were gathered from eight district hospitals, Army hospital Denpasar, and Sanglah central general hospital in Bali.	ELISA	[36]
2001–2004	A case control study used 94 confirmed JE cases and 163 aseptic meningitis non JE patients as control conducted to identify the determinants of close proximity to paddy fields and pig ownership.	ELISA	[38]
2005–2007	A total of 46 patients with JE confirmed and non JE encephalitis used to determine the association the risk of infection with paddy fields and the presence of pigs close to the patients houses.	ELISA	[37]
2015	17.7% of 282 Acute encephalitis and complex febrile leisure patients in Bali were confirmed infected by JEV in which 92% of them were children under 15 years old.	ELISA	[45]
Cases in international travellers in Bali			
1988	A ten-year-old girl from Australia had the JE infection on her 2-week holiday.	HI	[47]
1994	A Swedish woman traveller on 10 days holiday.	PRNT	[48]
1995	A Danish traveller aged 51 years old.	ELISA	[52]
2000	An 80-year-old Swedish man on 3 week holiday in Java and Bali.	IFA	[49]
2011	A 54-year-old Germany woman had spent a 2-week holiday.	IFA	[50]
2019	A 45-year-old Australian male spent 10 days in Bali in wet season.	Serological test	[51]
2019	A 59-year-old Australian male stayed for 3 months in Bali.	FL-MIA, ELISA, RT-PCR	[53]

HI: haemagglutination inhibition, ELISA: enzyme-linked immunosorbent assay, PRNT: plaque reduction neutralization test, IFA: immunofluorescence assay, FL-MIA: Flavivirus microsphere immunoassay, RT-PCR: reverse transcriptase polymerase chain reaction.

The reports on JEV-infected travellers in Bali may reveal the intensity of the viral transmission in the area. These traveller cases may also indicate that the virus could not only infect susceptible children but also adults who could reveal related clinical symptoms. To anticipate, the travellers have to prepare their selves having vaccinated against JEV before visiting the JE endemic area. In addition, related travel information has been widely provided online for the travellers to get the vaccine. However, some other international visitors, especially who came from non-JE endemic countries, tended to be vaccinated against JEV in Bali due to its lower price compared to the vaccine price in their own countries [54].

#### 3.2.3. Balinese social-cultural practices in relation to the potential risk of JEV circulation

An important factor that is likely associated with JEV-related cases in Bali is a high density pig population due to its socio-cultural practices. Although local economy and consumption may play less in the demand of local agricultural and livestock sectors, these factors, along with the socio-culture, are likely synergized in forming the risk. The Hindu community in Bali generally uses pigs for local investment, pork for meals and ceremonial requirements. A traditional ceremony called “*Tumpek Kandang”*, periodically held twice a year, is a local tradition offered by Balinese in regard to respect the ecological environment especially animals that live closely with humans [55]. Additionally, most of other traditional ceremonies in Bali in some cases required pigs per ceremony, making pig farming a key occupation [56]. It is estimated that the ratio of humans to pigs in Bali is 4:1 [33,44].

In addition, chickens and ducks are other animals that the Balinese also tend to rear as they are also required for the local ceremonial traditions, such as “*Caru*”, a procession of sacrificing animals in a ceremony in which chickens and ducks are commonly used [57]. Pork, ducks and chickens are not only offered in local Balinese ceremonies, they are also an important part of traditional local food consumption and a key protein source. A social community tradition, called “*Ngelawar”* is an activity implemented mostly by Balinese men to create a local specific type of food “*Lawar*”. It is a traditional food which requires minced meat of pork, ducks, or chickens to be mixed with some vegetables, coconut, herbs and spices ingredients. This traditional food is offered and consumed to the local community in a special ceremonial event [56].

**Figure2.**
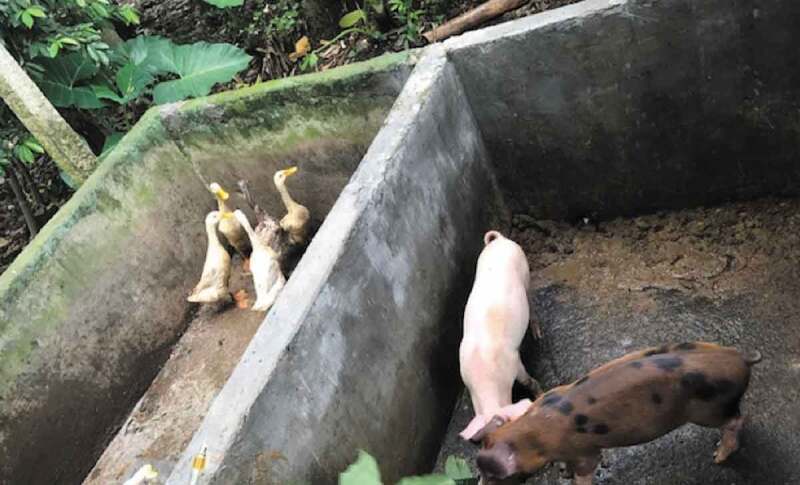
Small holder animal rearing practice in Bali. An example of keeping ducks next to the pigs (personal author's photo collection)

In regards to the local cultural practices, many local Balinese are raising pigs, chickens or ducks, along with managing their agricultural paddy fields. Some of them rear their ducks in their rice paddy at day time, while at dusk and night the ducks are kept next to their pigpens (Figure 2). In relation to the JEV transmission, these scale and intensity of agricultural and livestock production appear to be the drivers that contribute to the risk of JEV-intensive circulation. Consequently, the close proximity of the pig, chicken and or duck farming, rice paddy fields and the mosquitoes to the community is a key mix of risks for JEV to be amplified and transmitted to susceptible hosts, including humans.

It is really challenging to directly alter the local cultural practices in regards to minimize the risk of JEV exposure and infection. However, related local authorized staff should regularly conduct knowledge, attitude, and practice training program on JEV transmission control and prevention to the community, especially the local farmers. After the training, the community should realize the impacts of the disease on public health and livestock before practicing the JE prevention and control program in their lives. Then, the related knowledge, attitude and practice will gradually change the community’s habit before it becomes culturally accepted.

#### 3.2.4. JEV infection detected in animals in Bali

Surveys on JEV in animals in Bali are sporadic. The first survey was conducted in cattle in 1979 where 70% of 60 samples contained JEV antibodies [58]. Additionally, in 1993–1994, JEV antibodies were detected in 76.4% (107/140) of the pig samples, 95% (19/20) of horses, 43.6% (17/39) of cattle, and 23.8% (5/21) of goats [59]. From May to November 2003, a sentinel seroconversion survey was conducted using ten 2-month-old pigs and 8-month-old cattle in Denpasar, Bali. After eight months of observation, the antibodies were detected in 90% of the pig samples, while only 30% of the cattle seroconverted [60].

In addition, Widarso et al. [39] reported that in 1993–1994, antibodies against JEV were detected in 41.8% of 280 serums of pigs, cattle, goats and birds. The study was continued in 1996–1997, but only pig serum samples were assessed, with a seroprevalence of 46.7% (7/15) contained the antibodies [39]. However, in 2006, a serological survey performed in potential reservoirs of JEV, when the antibodies were identified lower at 32.2% (65/202) serum samples of pigs, including 20.7% (25/125) ducks and 36.7% (72/196) chickens [61].

Studies on detection of antibodies against JEV in animals in Bali were mainly focusing on the pigs as the potential reservoirs or amplifying hosts of the virus. Yamanaka et al. [62] detected the antibodies in 49% (60/123) of the pig samples collected from a subdistrict in Badung regency, Bali [62]. Moreover, a study demonstrated that all of the cattle serum samples collected in Denpasar city (54/54) contained the antibodies in comparison with Tabanan regency at 58.6% (17/29) and Buleleng regency at 47.8% (44/92). Whilst, in the pig serum samples tested, Tabanan had the highest antibody prevalence at 89.3% (25/28) compared to Denpasar at 66.13% (41/62) and Buleleng at 35% (21/60) [63]. More recently, Damayanti et al. [64] and Kuwata et al. [35] also conducted studies related to JE infection in pigs in Bali. The former performing the study in relation to determining the risk of JE infection in children [64], while the latter was to detect the genotype of the JEV circulated in Bali [35] (Table 2).

**Table 2.** Studies on antibodies against JEV detection in animals in Bali

Animal surveyed	Year of study	Findings	Test used	References
Cattle	1979	Antibodies against JEV detected in 70% of the samples.	HI	[58]
Pigs, cattle, goats, and avian	1993–1994	A total of 41.8% (117/280) serum samples were detected to contain the antibodies.	HI	[39]
Pigs, horses, cattle, goats.	1995	The antibodies against JEV were detected in 76.42% of pig serums, 95% horses, 43.6% cattle, and 23.8% goat serums.	HI	[59]
pigs	1996–1997	Antibodies against JEV were observed in 46.7% (7 out of 15) of pig serum samples.	HI	[39]
Cattle and pigs	2003	A sentinel study performed to detect seroconversion antibodies in each 10 of 8 months cattle and 2 months pigs. The antibodies against JEV were started to be detected in the week 3 of the study period in both animals.	ELISA	[60]
Pigs	2003	The antibodies were detected in 70% (280/400) of the collected pig serum samples.	HI	[37,44]
Cattle and pigs	2006–2007	Antibodies against JEV were detected in 77.14% (135/175) of the cattle serums and 58% (87/150) of pig serum samples collected in Denpasar city, Tabanan, and Buleleng regencies, Bali.	ELISA	[63]
Pigs, ducks, chickens	2006	The antibodies were detected in 32.2% (65/202) of pig, 20.7% (25/125) duck, and 36.7% (72/196) chicken serum samples.	ELISA	[61]
Pigs	2008	As many as 49% (60/123) of pig serum samples collected in Mengwi district, Badung regency, Bali were identified to contain the antibodies.	HI	[62]
Pigs	2015	A total of 60% (48/80) of pig serum samples were notified to have the antibodies (IgM & IgG) against JEV.	ELISA	[64]
Pigs	2017	Genotype IV of JEV was identified in 2 out of 5 isolates of 105 pig serums collected in Denpasar, Bali.	RT-PCR	[35]

HI: haemagglutination inhibition, ELISA: enzyme-linked immunosorbent assay, RT-PCR: reverse transcriptase-polymerase chain reaction.

The consistency of high seropositivity results reported from the animal sero-surveys, especially in pigs is important to be concerned as the animals could be the main sources of the virus circulating in the area. Through the mosquito vector, the JEV in the infected animals may be fed on by the vector and spread the virus to other susceptible hosts, including humans. In consequence, the high proportion of the antibodies detected in the animals may associate with the high number of JE cases in humans [65,66]. Further studies with proper sampling strategy and larger sample size are needed to evaluate more current prevalence in the pig population of Bali, including its economic impact to estimate the disease burden.

#### 3.2.5. JEV mosquito vector identification in Bali

In Bali, surveys on mosquitoes are somewhat limited, even in relation to their activity as vectors. Based on the related studies that have been performed, *Culex* spp. is the most dominant species found in Bali. In 1975, a survey was conducted to collect mosquitoes in the Indo-Australia archipelago for detecting potential mosquito vectors of arboviruses. Bali was a part of the survey area where *Anopheles barbirostris, Aedes vexans, Ae. albovictus, Cx. tritaeniorhynchus, Cx. gelidus, Cx. pipiens* and *Cx. pseudovishnui* were collected [40]. Furthermore, a 13-month study conducted to collect mosquitoes for assessing arthropod borne viruses in the south of Bali found 20 species of mosquitoes and several of them were potential JEV vectors, including *Cx. fuscocephala, Cx. gelidus, Cx. tritaenniorhynchus* [67]. *Cx. vishnui* [60] and *Cx. quinquefasciatus* [68] have also been identified on the island (Table 3).

**Table 3.** Reports on potential JEV mosquito vector in Bali

Year studies	Findings	References
1975	Potential JE vectors in Bali were collected, such as: Anopheles barbirostris, Aedes vexans, Ae. albovictus, Culex tritaeniorhynchus, Cx. gelidus, Cx. pipiens, Cx. pseudovishnui.	[40]
1980	Genotype IV was isolated from Cx. tritaeniorhynchus.	[34]
1983	Culex fuscocephala, Cx. gelidus, and Cx. tritaeniorhynchus that been incriminated to be involved in the Japanese encephalitis infection.	[67]
2004	Culex tritaeniorhynchus was the dominant mosquito trapped in Denpasar, Bali.	[60]
2015 2016	Survey was conducted in Jembrana regency, Bali and the JEV was detected in Cx. tritaeniorhynchus, Cx. vishnui, Cx. fuscocephala. Cx. tritaeniorrhynchus, Cx. gelidus, Cx. fuschophala could be found in Bali.	[13,37]
2018	Cx. tritaeniorhynchus was dominated mosquitoes trapped in the areas of Badung regency, Bali.	[68]

*Culex* spp. was dominated being collected in Bali as the potential mosquito vector of JEV.

Those findings proved that the *Culex* spp. mosquito, the main and primary vector of JEV can be found in Bali. In more specific, *Cx. tritaeniorhynchus, Cx. fuscochepala, Cx. gelidus, Cx. pseudovishnui and Cx. quinquefasciatus* that captured and identified in the area are the well-known mosquito species to be JEV vector [69]. However, the other species from different genera of mosquito found, such as *Anopheles* spp. and *Aedes* spp., were also reported to have a potential role in transmitting the JEV [70].

Those female adults of *Culex* spp. found are categorized to be nocturnal, zoophilic and anthropophilic mosquitoes. Instead of being more active at night [71], the mosquitoes found can not only feed blood from animals but from human as well. *Cx. tritaeniorhynchus, Cx. vishnue* and *Cx. gelidus* prefer to feed on pigs, cow, birds, including chickens. However, in the absence of those animal hosts, the mosquitoes can also feed on humans [69].

However, some of the *Culex* spp. mosquitoes have difference in their preferred habitats. The *Cx. tritaeniorhynchus, Cx. vishnue* and *Cx. gelidus* tend to be found in rural area, where agricultural paddy fields and pig farms are mainly established [15]. Nevertheless, the *Cx. quinquefasciatus* is more known to be urban mosquito. The mosquito prefers to breed in dirty stagnant water or polluted water bodies, for example in sewers, drains and ditches of the metropolitan area [16,72].

### 3.3. Other mosquito-borne viral diseases detected in Bali

Indonesia is a tropical country where some flaviviruses are circulating; however, the most frequent of the cases is dengue. Dengue fever is also a disease caused by a flavivirus, which can be fatal in infected humans and circulates endemically in the country [73,74], where Java and Bali islands reported as high infection regions. In Denpasar city Bali, the human cases of dengue in 2014 to mid of 2016 reached 6,898 cases with 38 fatal [75].

Other mosquito-borne viral diseases have also been detected in Bali, such as chikungunya and Zika viruses although they do not appear to be endemic. A total of 46.7% (7/15) of patients who suspected of being infected with chikungunya virus was confirmed by laboratory test results [76], while in patients who were suspected of Zika virus infection, only 7% (2/29) of them were confirmed [77] with the additional case report occurred in an international traveller [78]. Other related studies also demonstrated those infections detected in the area [74,79]. Meanwhile, West Nile, Murray Valley, St. Louis and yellow fever virus have never been reported.

Due to the endemic nature of dengue virus, cross reaction of antibodies with other flavivirus, like JEV does occur, affect the interpretation and validity of the diagnostic test results. To overcome this, testing is conducted against numerous antigens of the JEV serocomplex group to observe which has the highest titre in virus neutralization tests (VNTs) or plaque reduction neutralization tests (PRNTs). The tests are considered to be the gold standard of diagnostic techniques that can be used in the area where two or more flaviviruses circulate [80]. Alternatively, Western blotting or antigen protein microarray can also be used to identify the specific flavivirus antibodies [81].

### 3.4. Potential strategies for JE prevention and control program in Bali

Vaccination against JEV is considered an important strategy to control and minimize the disease impact on humans and livestock. Although it has not been implemented in animals in Indonesia, the vaccination against JEV in humans has been nationally applied. In the first quarter of 2018, Bali was chosen to be the first area in Indonesia that implemented the Chengdu SA-14-14-2 live-attenuated JEV vaccine targeted in 890,050 local children [33,82]. Almost 94% (95% CI: 92.8–94.9) of the children received the vaccination after the first campaign [83]. Although more studies needed to evaluate the vaccination program, it seems that the JEV infection in local children is getting lower.

Vaccination against JEV in animals is another consideration to prevent and minimize the infection in animals. Application of JE vaccination in pigs in Bali may minimize the virus transmission by decreasing the virus amplification as well as reducing the risk of the reproductive abnormalities in the animals, rate of the mosquito infection, and subsequently risk of transmission to humans. However, such a program would be difficult to be implemented. Although economic analysis on the related strategy need to be assessed, some reasons why the vaccination program in animals in Indonesia, including Bali has not been implemented, include the high cost of the vaccination, the high turnover of the susceptible pig population [84], and short period between maternal antibody waning (2–4 months) with the age of the pigs being slaughtered (6–7 months) [38], difficulty with cold chain and tracing, and farmers unwillingness to pay because their pigs do not get sick.

Public awareness is crucial, as the disease can be fatal for children and the elderly, with no specific antiviral treatment available to treat JEV infection [85]. A preventive program of JEV infection in public health is important to be applied, as it is a zoonotic disease that can impact on livestock and community. Lack of community knowledge, attitudes and practices (KAP) on the disease is a potential high risk for disease occurrence and spread to wider community [86]. Agricultural rice paddy field and pig farmers including their family members are likely in high risk to be exposed to the virus through the vector bites as they tend to be close contacted with the field and their animals.

Information and education on public to use bed-nets or insect-repellents as their personal protection measures along with implementation of a good sanitation may contribute to reduce contact with the mosquito vector. Similarly, housing the pigs indoor with anti-mosquito nets or screens especially during the peak of vector activity may minimize contact the mosquitoes to the animals [87,88]. Modification of rice paddy fields management for water and fertilizer usage and separating the paddy fields or preventing stagnant water around the pig farms are other strategies that may also limit contact between mosquitoes, pigs and humans.

JE is a vector-borne viral disease that requires to control the mosquito population to prevent the viral transmission and minimize the disease impacts. Limiting contact of the reservoir hosts and humans from mosquitoes or controlling the vector’s places for breeding may also support in reducing risk of the viral infection. These approaches are major strategies in controlling the JEV transmission. It is even more effective, when it combines with other related control strategies. Controlling the vector population integrated with public health, livestock and wild life managements is an important multisectoral measure for JEV control and prevention program in order to reduce the risk [89].

JEV infection occurred in the scope of human, environment and animals interaction, which also requires a multisectoral approach for controlling the disease. This approach has also been suggested to reduce and prevent the vector-borne disease [90]. Interaction in the integrated surveillance system is beneficial for not only being more understood of the disease happened but also assists early warning detection of the disease [91]. To apply this, related institutional and legal framework of the policy-makers should be more active in initiating, promoting and supporting the approach to be more effectively implemented [92].

## 4. Conclusion

Although the human JEV vaccination has just implemented, the high proportion of antibodies in the residents, the JE confirmed cases from acute encephalitis syndrome happened in children under 15 years old, and the related cases in international travellers recorded indicating high JEV transmission occurred in Bali. Socio-culture and ecology of local Balinese may be involved in the mosquito vector of *Culex* spp. abundance and high proportion of antibodies against JEV detected in the amplifying hosts and dominant livestock in the area. All of the interconnected components are likely contributed to the local viral circulation and consequent proportion of the JE related human cases in the area. This review has identified some key areas to concentrate on JE in Bali, including: a structured surveillance program in the animal reservoirs or livestock, identification of the viral characteristics circulated in the area and its other potential mosquito vector involved, estimation of the disease burden in affected humans and animals, and initiation of collaborative approach in understanding the disease occurrence in the ecological environment and socio-cultural interaction. By evaluating those key areas, it may contribute to better understand JEV transmission ecology and assist the prevention and control program in reducing the risk of the hyperendemic disease threat in the area of Bali.

## Data Availability

All data generated and analysed during this study are included in this manuscript.

## Geolocation information

The research location is in Bali province, Indonesia. The Bali’s latitude is −18.409518 and its longitude is 115.188919 with the GPS coordinates of 8° 24ʹ 34.2648” S and 115° 11ʹ 20.1084 E.

## References

[1] Filgueira L, Lannes N. Review of emerging Japanese encephalitis virus: new aspects and concepts about entry into the brain and inter-cellular spreading. Pathogens. 2019;8(3):111. 10.3390/pathogens8030111 PMC6789543 31357540

[2] Li N, Zhang Z-R, Zhang Y-N, et al. A replication-defective Japanese encephalitis virus (JEV) vaccine candidate with NS1 deletion confers dual protection against JEV and West Nile virus in mice. NPJ Vaccines. 2020;5(1):1–10. 32802412 10.1038/s41541-020-00220-4 PMC7406499

[3] Yu SP, Ong KP, Perera D, et al. Neuronal transcriptomic responses to Japanese encephalitis virus infection with a special focus on chemokine CXCL11 and pattern recognition receptors RIG-1 and MDA5. Virology. 2019;527:107–115. 30481615 10.1016/j.virol.2018.10.015

[4] Sehrawat S, Khasa R, Deb A, et al. Valosin-containing protein/p97 plays critical roles in the Japanese encephalitis virus life cycle. J Virol. 2021;95(11):e02336–20. 10.1128/JVI.02336-20 PMC8139707 33731458

[5] Choi J-W, Eom H-J, Kim HY, et al. Non-structural protein 1 from Japanese encephalitis virus expressed in E. coli retains its molecular weight and immunogenicity. Protein Expr Purif. 2020;169:105548. 31786309 10.1016/j.pep.2019.105548

[6] Turtle L, Solomon T. Japanese encephalitis—the prospects for new treatments. Nat Rev Neurol. 2018;14(5):298–313. 29697099 10.1038/nrneurol.2018.30

[7] Morita K, Nabeshima T, Buerano C, et al. Japanese encephalitis. Rev Sci Tech. 2015;34(2):441–452. 26601447 10.20506/rst.34.2.2370

[8] Pearce J, Learoyd TP, Langendorf BJ, et al. Japanese encephalitis: the vectors, ecology and potential for expansion. J Travel Med. 2018;25(Suppl_1):S16–S26. 29718435 10.1093/jtm/tay009

[9] Garjito TA, Prihatin MT, Susanti L, et al. First evidence of the presence of genotype- 1 of Japanese encephalitis virus in *culex gelidus* in Indonesia. BMC. 2019;12(19):1–4. 10.1186/s13071-018-3285-7 PMC6325860 30621763

[10] Li C, Di D, Huang H, et al. NS5-V372A and NS5-H386Y variations are responsible for differences in interferon α/β induction and co-contribute to the replication advantage of Japanese encephalitis virus genotype I over genotype III in ducklings. PLoS Pathog. 2020;16(9):e1008773. 32881988 10.1371/journal.ppat.1008773 PMC7494076

[11] Wei J, Wang X, and Zhang J, et al. Partial cross-protection between Japanese encephalitis virus genotype I and III in mice. PLOS Neglected Tropical Diseases. 2019;13(8):e0007601. 31374086 10.1371/journal.pntd.0007601 PMC6693775

[12] Karthika P, Vadivalagan C, Thirumurugan D, et al. DNA barcoding of five Japanese encephalitis mosquito vectors (*culex fuscocephala, culex gelidus, culex tritaeniorhynchus, culex pseudovishnui* and *culex vishnui*). Acta Trop. 2018;183:84–91. 29625090 10.1016/j.actatropica.2018.04.006

[13] Garjito TA, Widiarti AYM, Alfiah S, et al. Japanese encephalitis in Indonesia: an update on epidemiology and transmission ecology. Acta Trop. 2018;187:240–247. 30118700 10.1016/j.actatropica.2018.08.017

[14] Vargas RM, Korman TM, Nicholson S, et al. Shape relatedness between geographic populations of *culex tritaeniorhynchus*, the primary vector of Japanese encephalitis virus: a landmark study. Infect Genet Evol. 2021;90:104764. 33581329 10.1016/j.meegid.2021.104764

[15] Di Francesco J, Choeung R, Peng B, et al. Comparison of the dynamics of Japanese encephalitis virus circulation in sentinel pigs between a rural and a peri-urban setting in Cambodia. PLoS Negl Trop Dis. 2018;12(8). DOI:10.1371/journal.pntd.0006644. PMC6107123 30138381

[16] Nguyen-Tien T, Å L, Lindahl J, et al. Urban transmission of mosquito-borne flaviviruses–a review of the risk for humans in Vietnam. Infect Ecol Epidemiol. 2019;9(1):1660129. 31528273 10.1080/20008686.2019.1660129 PMC6735309

[17] Karna AK, Bowen RA. Experimental evaluation of the role of ecologically-relevant hosts and vectors in japanese encephalitis virus genotype displacement. Viruses. 2019;11(1):32. 10.3390/v11010032 PMC6356879 30621345

[18] Bae W, Kim JH, Kim J, et al. Changes of epidemiological characteristics of Japanese encephalitis viral infection and birds as a potential viral transmitter in Korea. J Korean Med Sci. 2018;33(9):e70. 29441740 10.3346/jkms.2018.33.e70 PMC5811662

[19] Pal M. Japanese encephalitis: a viral metazoonosis of growing public health importance. SF J Pub Heal. 2017;1(1):1–6.

[20] Ruget AS, Beck C, Gabassi A, et al. Japanese encephalitis circulation pattern in swine of northern Vietnam and consequences for swine’s vaccination recommendations. Transbound Emerg Dis. 2018;65(6):1485–1492. 29740970 10.1111/tbed.12885

[21] Mansfield KL, Hernandez-Triana LM, Banyard AC, et al. Japanese encephalitis virus infection, diagnosis and control in domestic animals. Vet Microbiol. 2017;201:85–92. 28284628 10.1016/j.vetmic.2017.01.014

[22] Henriksson E, Söderberg R, Ström Hallenberg G, et al. Japanese encephalitis in small-scale pig farming in Rural Cambodia: pig seroprevalence and farmer awareness. Pathogens. 2021;10(5):578. 34068673 10.3390/pathogens10050578 PMC8150308

[23] Kumar K, Arshad SS, Selvarajah GT, et al. Prevalence and risk factors of Japanese encephalitis virus (JEV) in livestock and companion animal in high-risk areas in Malaysia. Trop Anim Health Prod. 2018;50(4):741–752. 29243139 10.1007/s11250-017-1490-6 PMC5866273

[24] Bhattacharya S, Basu P. Japanese encephalitis virus (JEV) infection in different vertebrates and its epidemiological significance: a review. International Journal of Fauna and Biological Studies. 2014;1(6):32–37.

[25] Xiao C, Wang X, Cui G, et al. Possible pathogenicity of Japanese encephalitis virus in newly hatched domestic ducklings. Vet Microbiol. 2018;227:8–11. 30473356 10.1016/j.vetmic.2018.10.016

[26] Ladreyt H. Comparison of Japanese encephalitis force of infection in pigs, poultry and dogs in Cambodian Villages. Pathogens. 2020;9(9):719. 10.3390/pathogens9090719 PMC7558861 32882890

[27] Lord JS, Gurley ES, and Pulliam JRC, et al. Rethinking Japanese encephalitis virus transmission: a framework for implicating host and vector species. PLOS Neglected Tropical Diseases. 2015;9(12):e0004074. 26657648 10.1371/journal.pntd.0004074 PMC4686064

[28] Oliveira AR, Cohnstaedt LW, Noronha LE, et al. Perspectives regarding the risk of introduction of the Japanese encephalitis virus (JEV) in the United States. Front Vet Sci. 2020;7:48. 32118069 10.3389/fvets.2020.00048 PMC7019853

[29] Murhekar M, Vivian Thangaraj JW, Mittal M, et al. Acute encephalitis syndrome in Eastern Uttar Pradesh, India: changing etiological understanding. J Med Entomol. 2018;55(3):523–526. 29635529 10.1093/jme/tjy042

[30] Chow C, Dehority W. Long-term outcomes in children surviving tropical arboviral encephalitis: a systematic review. J Trop Pediatr. 2021;67(2):fmab028. 34109400 10.1093/tropej/fmab028

[31] Rawat PS, Patel K, and Mehrotra S, et al. Japanese encephalitis: a life threatening disease. Anusandhan-Vigyan Shodh Patrika. 2018;6(01):109–113.

[32] Gao X, Liu H, Li X, et al. Changing geographic distribution of Japanese encephalitis virus genotypes, 1935–2017. Vector-borne Zoonotic Dis. 2019;19(1). DOI:10.1089/vbz.2018.2291. 30207876

[33] Im J, Balasubramanian R, Yastini NW, et al. Protecting children against Japanese encephalitis in Bali, Indonesia. Lancet. 2018;391(10139):2500–2501. 29976465 10.1016/S0140-6736(18)31191-7

[34] Schuh AJ, Guzman H, Tesh RB, et al. Genetic diversity of Japanese encephalitis virus isolates obtained from Indonesian archipelago between 1974 and 1987. Vector-borne Zoonotic Dis. 2013;13(7). DOI:10.1089/vbz.2011.0870. PMC3700436 23590316

[35] Kuwata R, Torii S, Shimoda H, et al. Distribution of Japanese encephalitis virus, Japan and Southeast Asia, 2016–2018. Emerg Infect Dis. 2020;26(1):125. 31855535 10.3201/eid2601.190235 PMC6924880

[36] Kari IK, Liu W, Gautama IMK, et al. Clinical profiles and some associated factors of Japanese encephalitis in Bali. Paediatrica Indonesiana. 2006;46(1):13–19.

[37] Paramarta IGE, Kari IK, Hapsara S, et al. Faktor risiko lingkungan pada pasien Japanese encephalitis. Sari Pediatri. 2016;10(5):308–313.

[38] Liu W, Gibbons RV, Kari K, et al. Risk factors for Japanese encephalitis: a case-control study. Epidemiol Infect. 2010;138(9):1292–1297. 20109262 10.1017/S0950268810000063

[39] Widarso H, Purba W, and Suroso T, et al. Current status on Japanese encephlitis in Indonesia. In Annual Meeting of the Regional Working Group on Immunization in Bangkok, Thailand. 2002.

[40] Kanamitsu M, Taniguchi K, Urasawa S, et al. Geographic distribution of arbovirus antibodies in indigenous human populations in the Indo-Australian archipelago. Am J Trop Med Hyg. 1979;28(2):351–363. 453438 10.4269/ajtmh.1979.28.351

[41] Kari K. Japanese encephalitis at Sanglah central hospital, Denpasar. In 10th National Congress of Child Health, Bukittinggi, Indonesia. 1996.

[42] Yoshida M, Igarashi A, Suwendra P, et al. The first report on human cases serologically diagnosed as Japanese encephalitis in Indonesia. Southeast Asian J Trop Med Public Health. 1999;30(4):698–706. 10928363

[43] Campbell G, Hills SL, Fischer M, et al. Estimated global incidence of Japanese encephalitis: a systematic review. Bull World Health Organ. 2011;89(10):744–766. 10.2471/BLT.10.085233 PMC3209971 22084515

[44] Kari K, Liu W, Gautama K, et al. A hospital-based surveillance for Japanese encephalitis in Bali, Indonesia. BMC Med. 2006;4(1):1–7. 16603053 10.1186/1741-7015-4-8 PMC1481508

[45] Suwarba IGNM, Andayani AR, and Sukrata IW, et al. Japanese encephalitis incidence and its association with the length of stay and long-term outcome in 2015, Bali-Indonesia. Bali Medical Journal. 2016;5(1):135–137.

[46] Hills SL, Griggs AC, Fischer M, et al. Japanese encephalitis in travelers from non-endemic countries, 1973–2008. Am J Trop Med Hyg. 2010;82(5):930–936. 20439978 10.4269/ajtmh.2010.09-0676 PMC2861377

[47] Mac Donald WB, Tink AR, Ouvrier RA, et al. Japanese encephalitis after a two‐week holiday in Bali. Med J Aust. 1989;150(6):334–339. 2566108 10.5694/j.1326-5377.1989.tb136498.x

[48] Wittesjö B, Eitrem R, Niklasson B, et al. Japanese encephalitis after a 10-day holiday in Bali. Lancet. 1995;345(8953):856. 10.1016/s0140-6736(95)92990-8 7898243

[49] Rotzén Östlund M, Kan B, Karlsson M, et al. Japanese encephalitis in a Swedish tourist after travelling to Java and Bali. Scand J Infect Dis. 2004;36(6–7):512–513. 15307587 10.1080/00365540410020640

[50] Tappe D, Nemecek A, Zipp F, et al. Two laboratory-confirmed cases of Japanese encephalitis imported to Germany by travelers returning from Southeast Asia. J Clin Virol. 2012;54(3):282–285. 22465340 10.1016/j.jcv.2012.03.004

[51] Van K, Korman TM, Nicholson S, et al. Case report: japanese encephalitis associated with chorioretinitis after short-term travel to Bali, Indonesia. Am J Trop Med Hyg. 2020;103(4):1691–1693. 32783793 10.4269/ajtmh.19-0330 PMC7543813

[52] Buhl MR, Black FT, Andersen PL, et al. Fatal Japanese encephalitis in a Danish tourist visiting Bali for 12 days. Scand J Infect Dis. 1996;28(2):189. 8792489 10.3109/00365549609049074

[53] Pyke AT, Choong K, Moore F, et al. A case of Japanese encephalitis with a fatal outcome in an Australian who traveled from Bali in 2019. Trop Med Infect Dis. 2020;5(3):133. 10.3390/tropicalmed5030133 PMC7558094 32825150

[54] Wirawan I. Japanese encephalitis vaccine cost: a major reason to be vaccinated in Bali. J Travel Med. 2021. DOI:10.1093/jtm/taab050. 33772281

[55] Perni NN, and Sudarsana IK. Hindu religious education based on local wisdom in the Forest Area of Mandala Suci Wenara Wana Ubud. Journal of Critical Reviews. 2020;7(19):4975–4981.

[56] Sutisna P, Kapti IN, and Wandra T, et al. Towards a cysticercosis-free tropical resort island: a historical overview of taeniasis/cysticercosis in Bali. Acta Tropica. 2019;190:273–283. 30385216 10.1016/j.actatropica.2018.10.012

[57] Arnawa N, Gunartha IW, Sadwika IN, et al. Balinese hegemonic politeness in awig-awig of desa pakraman. Theory & Practice in Language Studies. 2018;8(11). DOI:10.17507/tpls.0811.13.

[58] Miura Y, Inaba Y, Tsuda Y, et al. A survey of antibodies to arthropod-borne viruses in Indonesian cattle. The Japanese Journal of Veterinary Science. 1982;44(6):857–863. 7182627 10.1292/jvms1939.44.857

[59] Ketut Santhia AP, Dibia N, and Eli Supartika K, et al. Prevalensi antibodi Japanese encephalitis di daerah Nusa Tenggara. Buletin Veteriner. 1996;IX(48): 1–10.

[60] Ketut Santhia AP, Putra AAG, Dibia N, et al. Surveilans terhadap Japanese encephalitis pada hewan sentinel. Buletin Veteriner. 2004;XVI(64): 69–74.

[61] Adi AAAM, Astawa NM, Damayanti PAA, et al. Seroepidemiological evidence for the presence of Japanese encephalitis virus infection in ducks, chickens, and pigs, Bali-Indonesia. Bali Medical Journal. 2016;5(3): 533–537.

[62] Yamanaka A, and Mulyatno KC, Susilowati, H, et al. Prevalence of antibodies to Japanese encephalitis virus among pigs in Bali and East Java, Indonesia, 2008. Japanese Journal of Infectious Disease. 2010;63:58–60. 20093765

[63] Ketut Santhia AP, Dibia N, Putra AAG, et al. Seroprevalensi Japanese encephalitis pada sapi dan babi di kota Denpasar, Kabupaten Buleleng dan Tabanan. Buletin Veteriner. 2008;XX(73): 83–88.

[64] Damayanti PAA, Adi AAAM, Astawa INM, et al. Incidence of Japanese encephalitis among children is associated with the presence of pigs in Bali, Indonesia. Biomedical and Pharmacology Journal. 2017;10(3). DOI:10.13005/bpj/1237.

[65] Dhanze H, Kumar MS, Singh V, et al. Detection of recent infection of Japanese encephalitis virus in swine population using IgM ELISA: a suitable sentinel to predict infection in humans. J Immunol Methods. 2020;486:112848. 32891615 10.1016/j.jim.2020.112848

[66] Baruah A, Hazarika RA, Barman NN, et al. Mosquito abundance and pig seropositivity as a correlate of Japanese encephalitis in human population in Assam, India. J Vector Borne Dis. 2018;55(4):291. 30997889 10.4103/0972-9062.256564

[67] Lee VH, Atmosoedjono S, Rusmiarto S, et al. Mosquitoes of Bali Island, Indonesia: common species in the village environment. Southeast Asian J Trop Med Public Health. 1983;14(3):298–307. 6140760

[68] Ambarawati IGAA, Adi AAAM, and Damayanti PAA, et al. Knowledge and prevention of farmer households to the Japanese encephalitis infection in Badung Regency, Bali Province, Indonesia. Advances in Social Sciences Research Journal. 2020;7(10):37–48.

[69] Boyer S, Durand B, Yean S, et al. Host-feeding preference and diel activity of mosquito vectors of the Japanese encephalitis virus in Rural Cambodia. Pathogens. 2021;10(3):376. 33800999 10.3390/pathogens10030376 PMC8003966

[70] Kim DM, Noh BE, Heo J, et al. Seasonal prevalence of mosquitoes collected from light traps in Gyeongsangnam province, Republic of Korea (2013–2014). Entomological Research. 2018;48(5): 437–445.

[71] Bashar K, Sarker A, Asasuzzaman M, et al. Host preference and nocturnal biting activity of mosquitoes collected in Dhaka, Bangladesh. International Journal of Mosquito Research. 2020;7(3): 1–8.

[72] Bordoloi B, Saharia S. Mosquito-borne diseases in Assam. International Journal of Mosquito Research. 2021;8(2):130–133.

[73] Masyeni S, Yohan B, Somia IKA, et al. Dengue infection in international travellers visiting Bali, Indonesia. J Travel Med. 2018;25(1):1–7. 10.1093/jtm/tay061 PMC6118167 30113689

[74] Dhewantara PW, Marina R, Puspita T, et al. Spatial and temporal variation of dengue incidence in the island of Bali, Indonesia: an ecological study. Travel Med Infect Dis. 2019;32:101437. 10.1016/j.tmaid.2019.06.008 31362115

[75] Hamid PH, Prastowo J, Widyasari A, et al. Knockdown resistance (kdr) of the voltage-gated sodium channel gene of aedes aegypti population in Denpasar, Bali, Indonesia. Parasit Vectors. 2017;10(1):283. 28583207 10.1186/s13071-017-2215-4 PMC5460344

[76] Sari K, Myint KSA, Andayani AR, et al. Chikungunya fever outbreak identified in North Bali, Indonesia. Trans R Soc Trop Med Hyg. 2017;111(7):325–327. 29029262 10.1093/trstmh/trx054 PMC7064274

[77] Sasmono RT, Johar E, Yohan B, et al. Spatiotemporal heterogeneity of zika virus transmission in Indonesia: serosurveillance data from a pediatric population. Am J Trop Med Hyg. 2021;104(6):2220. 10.4269/ajtmh.21-0010 PMC8176489 33939632

[78] Leung G, Baird RW, Druce J, et al. Zika virus infection in Australia following a monkey bite in Indonesia. Southeast Asian J Trop Med Public Health. 2015;46(3):460–464. 26521519

[79] de Jong W, Rusli M, Bhoelan S, et al. Endemic and emerging acute virus infections in Indonesia: an overview of the past decade and implications for the future. Crit Rev Microbiol. 2018;44(4):487–503. 29451044 10.1080/1040841X.2018.1438986

[80] Nurtop E, Villarroel PMS, Pastorino B, et al. Combination of ELISA screening and seroneutralisation tests to expedite zika virus seroprevalence studies. Virol J. 2018;15(1):1–6. 30587193 10.1186/s12985-018-1105-5 PMC6307276

[81] Garg H, Mehmetoglu-Gurbuz T, Joshi A, et al. Virus like particles (VLP) as multivalent vaccine candidate against chikungunya, Japanese encephalitis, yellow fever and zika virus. Sci Rep. 2020;10(1):1–13. 32132648 10.1038/s41598-020-61103-1 PMC7055223

[82] Sawitri AA, Yuliyatni PC, Ariawan MD, et al. Limitations of immunization registers at community health centers for measuring immunization coverage: a case study of the Japanese encephalitis mass immunization program in Bali Province, Indonesia. Osong Public Health Res Perspect. 2021;12(3):158–168. 34102049 10.24171/j.phrp.2020.0241 PMC8256298

[83] Suardani N, Wirawan DN, and Sawitri A, et al. The role of information sources and characteristics of children in the acceptance of Japanese encephalitis (JE) mass immunization in Bali Province. Public Health and Preventive Medicine Archive. 2019;7. 75–84.

[84] Ladreyt H, Durand B, Dussart P, et al. How central is the domestic pig in the epidemiological cycle of Japanese encephalitis virus? A review of scientific evidence and implications for disease control. Viruses. 2019;11(10):949. 10.3390/v11100949 PMC6832429 31618959

[85] Wei J, Hameed M, and Wang, X. Antiviral activity of phage display-selected peptides against Japanese encephalitis virus infection in vitro and in vivo. Antiviral Res. 2020;174():104673. 31812636 10.1016/j.antiviral.2019.104673

[86] Singh BB, Kaur R, Gill GS, et al. Knowledge, attitude and practices relating to zoonotic diseases among livestock farmers in Punjab, India. Acta Trop. 2019;189:15–21. 30268685 10.1016/j.actatropica.2018.09.021

[87] Karthikeyan A, Shanmuganathan S, Pavulraj S, et al. Japanese encephalitis, recent perspectives on virus genome, transmission, epidemiology, diagnosis and prophylactic interventions. J Exp Biol Agric Sci. 2017;5(6):731–748.

[88] Yeo G, Chan S, How CB, et al. Molecular analysis of the bloodmeals of culex spp. mosquitoes at natural habitats in Singapore to investigate the potential risk of Japanese encephalitis virus and West Nile Virus transmission. Vector-borne Zoonotic Dis. 2020;20(9):703–714. 32931404 10.1089/vbz.2019.2576

[89] Yap G, Mailepessov D, Lim XF, et al. Detection of Japanese encephalitis virus in *culex* mosquitoes in Singapore. Am J Trop Med Hyg. 2020;103(3):1234. 32700679 10.4269/ajtmh.19-0377 PMC7470584

[90] WHO. Multisectoral approach to the prevention and control of vector-borne diseases: a conceptual framework (World Health Organization, Geneva). 2020.

[91] Johnson I, Hansen A, Bi P, et al. The challenges of implementing an integrated one health surveillance system in Australia. Zoonoses Public Health. 2018;65(1):e229–e236. 29226606 10.1111/zph.12433 PMC7165821

[92] Acharya KP, Karki S, Shrestha K, et al. One health approach in Nepal: scope, opportunities and challenges. One Health. 2019;8:100101. 31485475 10.1016/j.onehlt.2019.100101 PMC6715885

